# Science system path-dependencies and their influences: nanotechnology research in Russia

**DOI:** 10.1007/s11192-016-1916-3

**Published:** 2016-04-01

**Authors:** Maria Karaulova, Abdullah Gök, Oliver Shackleton, Philip Shapira

**Affiliations:** Manchester Institute of Innovation Research, Alliance Manchester Business School, University of Manchester, Manchester, UK; Institute of Management, Scuola Superiore Sant’Anna, Pisa, Italy; School of Public Policy, Georgia Institute of Technology, Atlanta, GA USA

**Keywords:** Russia, Nanotechnology, Path dependence

## Abstract

In this paper, we study the influence of path dependencies on the development of an emerging technology in a transitional economy. Our focus is the development of nanotechnology in Russia in the period between 1990 and 2012. By examining outputs, publication paths and collaboration patterns, we identify a series of factors that help to explain Russia’s limited success in leveraging its ambitious national nanotechnology initiative. The analysis highlights four path-dependent tendencies of Russian nanotechnology research: publication pathways and the gatekeeping role of the Russian Academy of Sciences; increasing geographical and institutional centralisation of nanotechnology research; limited institutional diffusion; and patterns associated with the internationalisation of Russian research. We discuss policy implications related to path dependence, nanotechnology research in Russia and to the broader reform of the Russian science system.

## Introduction

In recent years, national governments in developed and developing countries have often sought to optimise their science systems, for example, by invoking large-scale reforms (Huang et al. 2015), by implementing research assessment programmes (Weingart 2005), or by changing mechanisms of funding to incentivise priority research areas (Roco 2011). At the same time, national science policies have increasingly pursued goals to target frontier or emerging technologies, such as nanotechnology or synthetic biology, that promise new economic and competitive advantages and new capabilities to meet societal and environmental challenges (Hullmann 2006; Shapira and Wang 2010).

Policy ambitions to simultaneously improve efficiency and performance yet also to address emerging research areas present major challenges for science systems. This is particularly the case when emerging research areas require not only additional resources but also new methods and interdisciplinary collaborations. From an evolutionary perspective (Dosi and Nelson 1994), the relative performance of reforms and policy initiatives on emerging technologies depends on current as well as past features of the system. Rapid and visible change is frequently demanded by policy makers, yet research systems and their constituent scientific institutions and practices are typically slow, or even resistant, to give up established ways. Path dependence—where institutional forms and practices formed in earlier periods persist and disproportionately influence current activities and trajectories—is potentially an underpinning factor that not only shapes change, but which can also inhibit progress through lock-in and negative feedback loops (Nelson and Winter 1982; Schienstock 2007). In this paper, we study the influence of path dependencies on the development of an emerging technology in a transitional economy. We use this case to examine the path-dependent factors that limit research system change and subsequent performance.

Our focus is the development of nanotechnology^1^ in Russia from 1990 to 2012. Following the disbanding of the Soviet Union in 1991, Russia has experienced a series of political and economic crises that profoundly impacted its research capabilities. From the 1990s through to the present, Russian scientific research has remained severely underfunded: over the last decade, R&D spending as a share of the Gross Domestic Product has plateaued at the level of about 1.1 % (UNESCO 2015). This has been accompanied by high rates of scientific out-migration (Graham and Dezhina 2008), when Russia lost nearly one-quarter of its authorship share of the world’s scientific articles between 1996 and 2008 (The Royal Society 2011). Nevertheless, Russia has well-established capabilities in nanoscience and its underlying disciplines (including physics, chemistry, materials science, and electronics). By encouraging nanoscience research, the Russian government hoped to leverage Russia’s position back to the top and join the leading countries in terms of nanotechnology research and commercialisation. This was also expected to bolster the indigenous science, technology and innovation system.

Russia has been reforming its research system throughout the post-Soviet period, including ambitious projects to build a more effective system of innovation (OECD 2011). However, Soviet-style institutional models (Fortescue 1992), especially within the Academy of Sciences, have persisted. In fact, although the organisation of science was broadly perceived as inefficient within Russia, government-led science system reforms did not start in earnest until the late 2000s. To date, Russia’s nanotechnology initiatives have not yielded the desired results. Whereas China is lauded as a successful case of using emerging technologies to catch up with the leaders in terms of publication outputs (Arora et al. 2013), Russia has lagged. Russia ranked sixth in annual nanotechnology publication outputs in 1990, but dropped to tenth place by 2010 (Terekhov 2012). In the 1990s and 2000s, the growth of nanotechnology papers and patents was significantly lower in Russia than in China and India (Liu et al. 2009).

### Research objectives

It has been suggested that Russia’s efforts in leveraging its science and technology outputs have been hindered by ‘stubborn path-dependencies’ (Klochikhin 2012). Russia’s recent designs to build a competitive basis for nanotechnology research provide a lens through which we can examine the extent to which the Russian science system has (or has not) developed. This research is based on bibliometric analyses to identify path-dependent practices, patterns, and institutions in Russian nanoscience, that appear to be resistant to reform. We build on a line of work that includes an earlier overview of Russia’s profile in nanotechnology in the post-Soviet period (Karaulova et al. 2014) and comparative research, which examines post-Socialist developments in science and innovation in China and Russia (Klochikhin 2013; Klochikhin and Shapira 2012).

The main objective of this study is to link bibliometric performance indicators of Russian nanoscience and nanotechnology with path-dependent elements of the institutional set-up of its research system. Previous studies suggest that institutional constraints have an impact on science indicators, for example, international collaboration (Wagner and Leydesdorff 2005). In order to further elucidate this, we employ a country-report framework of bibliometric analysis of nanotechnology performance: main actors (institutional and individual), publication outlets, co-authorship patterns, quality of research outputs, geography of research, and the structure of international collaboration (Glanzel 1996; Moed et al. 1995; Tang and Shapira 2011). Structural changes over time are important for tracing sustained systemic features as well as institutional rigidities.

We first consider the main institutional actors of Russian nanotechnology and find among them a seemingly overwhelming domination of the Russian Academy of Sciences (RAS) in terms of the quantity of outputs, but also in terms of the concentration of collected citations and top performing scientists. Further, we consider the persistence of journal gateways, where publications are issued in translated versions of Russian, rather than internationally published, journals. We then examine the on-going geographic centralisation of Russian nanotechnology research and its multi-layered qualities. It is shown that Moscow and the Moscow region are foremost in publications over other previously strong centres of St. Petersburg and Novosibirsk. We find that leading research in terms of star scientists is also centralised within RAS. We then turn to institutional diffusion, highlighting how Soviet-era practices of organisational segregation of researchers from different institutes and disciplinary traditions have persisted in more recent Russian domestic research. Finally, we examine the structure of internationally collaborated publications of Russia, highlighting the plateauing trend of collaboration rates with the USA alongside with the increasing role of other post-Soviet states.

The paper is structured as follows: The next section provides a background review, highlighting literature on the concept of path dependence and discussing what available bibliometric and other research says about the Russian science system and the development of nanotechnology. The following section focuses on methodology and data. A results section provides an updated overview of the Russian research system and then focuses on elements of nanotechnology research that highlight path-dependent elements of scientific knowledge production in Russia. The conclusions outline the main findings and policy recommendations, as well as limitations and directions for future research.

## Background review

### Path dependence

In discussions of technological choices, path dependence has been used to characterise circumstances where a system or an object of analysis was at a point of divergence and chose a particular technological trajectory which has persisted even as more efficient technologies emerged (Liebowitz and Margolis 1995). We use path dependency to also encompass institutions and practices—as a “social process” that refers to “causal relevance of preceding events in a temporal sequence” (Pierson 2000). Not switching to a more favourable alternative is a signal of path dependence, although how these situations emerge in social and innovation processes is problematic (Martin and Sunley 2006). The temporal dimension is also important: institutional evolution needs to be seen in long-term perspective.

Demonstrating persistent path dependencies in scientific activities and performance is a challenging task: although it can be aided by the use of bibliometric tools, only a few bibliometric research papers have used the concept explicitly. Path dependence is usually interpreted in terms of trajectories, regimes or historically inherited advantages or disadvantages, which condition dynamics of publication and patent outputs (Radosevic and Yoruk 2014). These notions are deeply rooted in the evolutionary tradition of scientometric analysis (Leydesdorff 2013). Such an approach reveals trajectories (paths) pursued by regions, countries, institutions, organisations or individuals. Being path-dependent for each unit of analysis in this context means being locked in a particular track or line of development.

We suggest that examination of key events and policy interventions provides further opportunities for bibliometric study to offer insights and hints as to causation of path-dependent systems. In the Russian case, a succession of major system-wide events could be expected to have influenced the direction and performance of the science system. These include the breakup of the Soviet Union, innovation system-building activities in the early-to-mid 2000s, and efforts to reform science in the late 2000s. The example of nanotechnology research in Russia allows us to see what has changed in terms of institutional roles and practices during this tumultuous period, revealing both specific and broader insights about path dependence in the Russian science system.

### Russian science, technology, innovation and nanotechnology

From the early 1990s to the mid-2000s, there were few explicit policy implementation linkages in Russia between technology and economic growth. During this period, Russia was engaged primarily in large-scale economic transformation and institutional building (Gianella and Tompson 2007). Russian policy interest in the potential of nanotechnology to drive new technology-based growth followed some years after the establishment of national nanotechnology programmes in the United States and other developed and developing countries. In particular, China was starting to challenge US domination in science and technology by using nanotechnology as leverage (Kostoff 2008). In Russia, nanotechnology was identified as a priority research area for the first time in 2004 (The Government of Russia 2004), while in 2007 the Russian government launched its own national nanotechnology initiative. The announcement of plans to invest about $11 billion in nanotechnology in Russia through to 2015, and the formation of Rusnano—a government company charged with commercialising nanotechnology, generated much interest, hope, and some scepticism the domestic and international observers (Nature 2009; Schiermeier 2007, 2009). For Russia, the national nanotechnology initiative was a political as well an economic, scientific and technological project. Russia emerged as one of the global leaders in government-led nanotechnology investment, reaching about $1 billion per year (Lux Research 2013).

In tandem, the Russian government accelerated its efforts to reform the science, technology and innovation system. A recent policy iteration emphasises ‘Institutes of Development’ (Ministry of Economics of Russia 2015), which are designed to act as ‘innovation lifts’ in scaling up emergent companies and science-intensive start-ups. Nanotechnology is one of the components of this programme, with Rusnano, now reorganised as a venture company, designated as one of the ‘Institutes of Development’. However, implementation issues have emerged. It has been argued that the Russian government over-estimated the ability of the Russian science system to deliver commercialisable research (Terekhov 2013). Studies have suggested limited evidence of progress by Russian agencies and industries in creating domestic markets for nanotechnology, in making Russian nanotechnology products internationally competitive (Gokhberg et al. 2012) or in meeting expectations (European Commission 2013). Other science system reforms, such as the transition to a grant-based funding procedures and the reform of the Academy of Sciences, were not launched until 2012.

### Russian nanotechnology: national and international comparative perspectives

Nanotechnology has gathered significant interest from bibliometric research since the early 2000s after the United States and China adopted large-scale policy and funding programmes to invest heavily, to stimulate scientific development and interdisciplinary research (Shapira et al. 2015). China’s position as an emerging county and its significant increase in research outputs has made it a frequent focus for analysts (Appelbaum et al. 2011; Bhattacharya and Bhati 2011). However, Russia has received less attention by the international research community. Some studies compare the performance of Russia in nanotechnology in the context of other emerging economies, such as China, India or Brazil. Russia is depicted as a country that invested much in nanotechnology, but lost momentum in publication and patent outputs, effectively growing relatively slowly and with fewer scientific, innovation, and economic returns (Liu et al. 2009, 2011; Wong and Wang 2015). Other studies reported similar results for scientific outputs in general: Russia has been reported to be the worst-performing BRICS country in terms of expected contribution of top-cited papers (Bornmann et al. 2015).

With regard to its national features, Russian science remained very closed until the breakdown of the Soviet Union. After 1990 the country opened to the world, which resulted in the explosive growth of international collaborations (Glänzel et al. 1999). Russia also maintained an important position in international collaboration networks of Eastern European countries (Kozak et al. 2015). Within its national borders, however, the institutional setting of Russian science was relatively unchanged: the Russian Academy of Sciences (RAS), the successor of the Soviet Academy, remained the main research performing organisation across the sciences, second only to the Chinese Academy of Sciences in its gross annual publication output (Kostoff et al. 2008). The science funding system remained highly centralised, and universities maintained mainly teaching specialisation until mid 2000s (Graham and Dezhina 2008). The domination of RAS is maintained across most fields and disciplines of Russian science, with an exception of clinical medicine, in terms of the volume of outputs and collected citations (Markusova et al. 2009a, b; Mokhnacheva and Kharybina 2011). Some authors equate RAS research outputs with overall research outputs of Russia (Markusova et al. 2009a, b).

The entire system has remained heavily skewed towards natural sciences, mainly, physics, where leading research is concentrated (Glanzel 2001; Kotsemir 2012; Pislyakov and Shukshina 2014; Wilson and Markusova 2004). Zitt and Bassecoulard (2004) report weak involvement of Russia and other post-Soviet countries in life sciences. Russian nanotechnology maintains the focus on physics (Liu et al. 2011). Existing bibliometric research indicated several commonly reported strengths and weaknesses that Russian nanotechnology shares with other disciplines. These are: underdeveloped nanoscience infrastructure (Connolly 2013), bureaucracy (Yaminsky 2006), aging research-performing personnel and other human resource issues (Terekhov 2011), and problems with political priorities in nanotechnology research and commercialisation (Terekhov 2012). At the same time, existing scholarship notes the relative strength of Russian nanotechnology in terms of fundamental approaches and strong legacy of Soviet research (Andrievski 2003), and identifies areas where Russia is not only internationally competitive, but also has the potential to become a world leader, such as fullerenes and nanodiamond research (Terekhov 2015).

The domestic nanotechnology strategy of Russia has therefore differed from strategies of other developing countries due to its unique situation. Instead of relying on internationalisation and attracting leading researchers from abroad like China (Klochikhin and Shapira 2012), the Russian government put its stakes on supporting existing competitive nanotechnology areas and investing heavily in infrastructure of research and development (Terekhov 2013). However, this has not yielded the desired results, and Russia’s international standing in nanotechnology has deteriorated throughout the first decade of the twenty-first century. Relatively few internationally authored bibliometric papers delve more deeply to investigate the issues behind this apparent systemic failure. In contrast, work by Russian researchers has been mostly focused on a domestic critical discourse.

We note that recent reforms to the Russian science system (initiated from 2013) were partly in reaction to the underperformance of Russia’s flagship national nanotechnology initiatives. In this paper, we not only provide an up-to-date bibliometric analysis of nanotechnology research in Russia, but also link this to an analysis of structural aspects of the Russian science system. As nanotechnology is not a cohesive research field, but rather spans across the disciplines, encompassing leading and interdisciplinary research areas, the findings of this study are important to understand systemic problems within best-performing areas of Russian research. This addresses the necessity of further reflection on the challenges posed by deeply embedded path-dependent inefficiencies in the Russian science system.

## Data and methodology

This research employs a mixed-method design. On the quantitative side, we conducted a bibliometric analysis of scientific publications by Russian authors in nanotechnology. On the qualitative side, we completed a series of 24 semi-structured interviews in Moscow in March and April 2014. Each interview lasted from 45 min to several hours, and the scope of interviewees included small and large companies, federal and regional government officials, and university and Academy of Sciences researchers.

The bibliometric dataset covers the time period from 1990 to 2012 and spans three phases of Russian nanoscience development: the transitional period after the breakup of the Soviet Union (1990–2004); the development of the Russian national nanotechnology initiative (2005–2007); and the recent period of nanotechnology research (2008–2012). We utilised the Web of Science (WoS) by Thomson Reuters as the main data source. Publications where at least one author had a Russian affiliation address (the Soviet Union in 1990–1992) were identified as Russian publications. The primary language of publications in the dataset is English, but it includes translated Russian journals and specialised editions with translated articles originally published in Russian.

Science in the Soviet Union developed in parallel, but not always in cooperation, with researchers elsewhere in the world. This is a practice subsequently continued in the Russian Federation. Research in areas related to nanoscience started developing in the Soviet Union *en masse* as early as the 1980s (Terekhov 2013). However, use of the terminology of ‘nanoscience’ and ‘nanotechnology’ was not common until these expressions entered the Russian policy and funding landscape in the early 2000s. To accommodate this, we use a definitional approach to nanotechnology that incorporates a range of relevant and related terms and then subsequently removes extraneous items. This two-stage lexicological query is detailed in Porter et al. (2008) and updated in Arora et al. (2013). The first stage applies a keyword search based on Boolean queries. In the second stage, unrelated records are removed by applying exclusion terms.

This paper examines scientific publication patterns (paths and trajectories) of the Russian nanotechnology actors using their authorship and co-authorship data. While co-authorship is not the only form that collaboration takes (Bozeman and Corley 2004), it has become an established metric, which has a long tradition of use in bibliometric scholarship (Barabasi et al. 2002; Newman 2004).

Institutional authorship of nanotechnology publications is used to track scientific outputs of research organisations over time, and study the channels of these outputs, such as scientific journals. The citations of articles authored by top Russian scientists are used to examine clustering of excellent research within organisational units. Co-authorship data, measured as co-occurrence of different affiliation addresses in one publication, is used to measure intra-national collaboration density in Russia. International co-authorship, measured as co-occurrence of two countries in publication address data (Glanzel 2001) is used to examine patterns of Russian international collaborations.

After the publication data was collected and refined, further data cleaning to remove duplicates and consolidate organisational and author names was undertaken. The data was processed pursuing strategies of aggregation and disambiguation. Problems of aggregation include affiliation, location, funding source, and author categories that the database recognises as separate due to spelling and translation issues. Disambiguation problems relate to similarly named entities, such as organisational affiliations and author names, which had been merged together. Particular effort was put into separating and aggregating institutes of the Russian Academy of Sciences. RAS is a large research organisation that comprises more than 500 research institutes, of which 261 published in nanotechnology in the 1990–2012. RAS is distinctive in the Russian science system as a national research entity with centralised governance and budget allocations, yet where each institute performs autonomously and with varying specialties and outputs. Creating fields, which distinguished individual RAS institutes, scientific centres and laboratories, yielded additional value.

We further grouped the data according to author country, region, and type of affiliation. In addition to distinguishing universities and Academy of Science institutes, we also identified public research organisations. This category includes private and state-owned research institutes that are not associated with universities or RAS. We further identified corporate actors—this category comprises private and state-owned companies that have a distinctive property type label in their names (including LLC, Ltd, GmbH, and ZAO). Other organisations included those that could not be attributed to any other category. Finally, to examine the internationalisation of Russian science we also separated publications into non-internationally collaborated publications (NCP) and internationally collaborated publications (ICP). The two groups are mutually exclusive. NCP are authored by single or multiple researchers only with domestic Russian affiliations. ICP are authored by researcher combinations with Russian and international affiliations. Following the search and cleaning process, a total of 33,538 Russian WoS nanotechnology publication records were identified between 1990 and 2012 that were produced by 1512 unique organisations.

We also conducted network analysis to reinforce the quantitative side of the methodology. This included employing network visualisations in which nodes represent author affiliation organisations and edges as the co-authorship between these organisations and also a number of social network statistics. Our network analysis covered the full 1990–2012 period as well as the three phases of Russian nanoscience development mentioned above in this Section.

Software suites employed include VantagePoint v9.0 for data consolidation, cleaning and analysis and Gephi v0.82 and v0.9 for network analysis and visualisation.

## Results

**Fig. 1:**
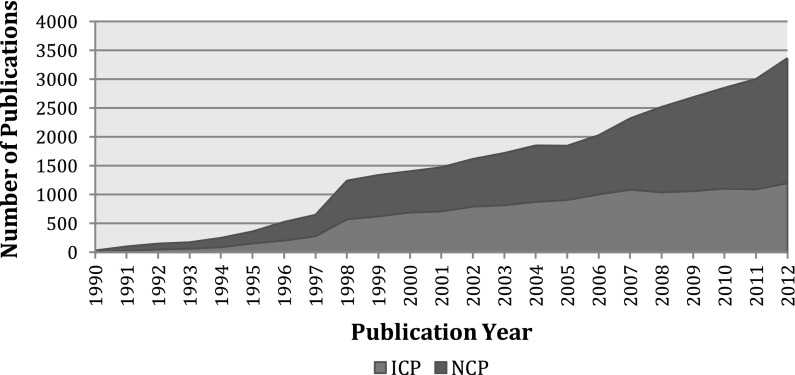
Russian nanotechnology publications, by collaboration type, 1990–2012. *Source*: Web of Science. See text for details. *Note*: *ICP* internationally collaborated publications, *NCP* non-internationally collaborated publications

A review of the publication records broadly indicates trends in the types of Russian nanotechnology publications. The annual output of Russian nanotechnology publications steadily increased between 1990 and 2012. In 1998, there was a considerable jump in the number of publications. This probably reflects the inclusion of additional Russian journals in the WoS. Growth rates for domestic and international publications are almost identical starting from 1999 until 2012 and are about 1.1 % per year. On average, domestic publications grow 2 % faster than internationally collaborated publications (Fig. 1).

Physics is the dominant subject category in the disciplinary structure of nanotechnology output, constituting over a half of all publications, but decreasing from 72 % in 1991 to 52 % in 2012. Two-thirds of publications are written by authors affiliated with RAS, university authors are associated with around 40 % of publications, and around 10 % of publications include authors from public research organisations (Table 1). Around 43 % of publications include a non-Russian author. The internationalisation of publications as measured by percentage of publications across different organisations including a non-Russian affiliation does not greatly vary from this mean for all nanotechnology publications.

**Table 1:** Russian nanotechnology publications by organisation types, 1990–2012

	Number of organisations	Number of publications	Share of all publications (%)	Share of ICP (%)
All Russian organisations	1512	33,528	100.0	43.0
Academy of sciences	261	22,794	68.0	43.0
University	396	13,868	41.4	38.3
PROs	432	3781	11.3	44.9
Corporate	420	982	2.9	30.1
Other	3	3	0.0	33.3

In addition to the Russian Academy of Sciences there are two other Academies in Russia: the Russian Academy of Medical Sciences (contributed 190 publications) and the Russian Academy of Agricultural Sciences (14 publications). ICP = Internationally Collaborated Publications

*Source* Web of Science. *N* = 33,528 publication records. See text for details

**Fig. 2:**
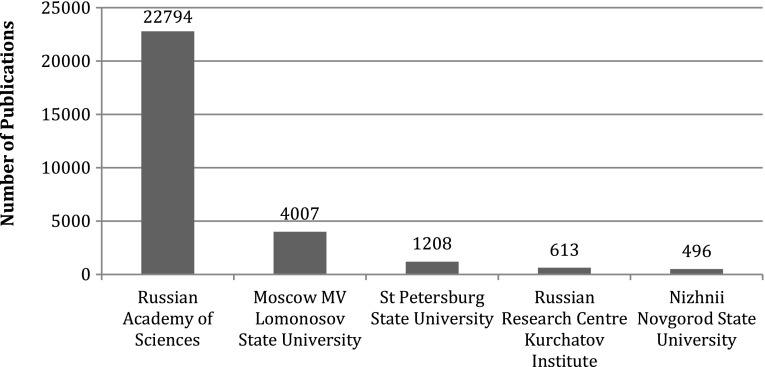
Foremost publishers in Russian nanoscience, 1990–2012. *Source*: Web of Science. See text for details. *N* = 33,538 publication records

**Fig. 3:**
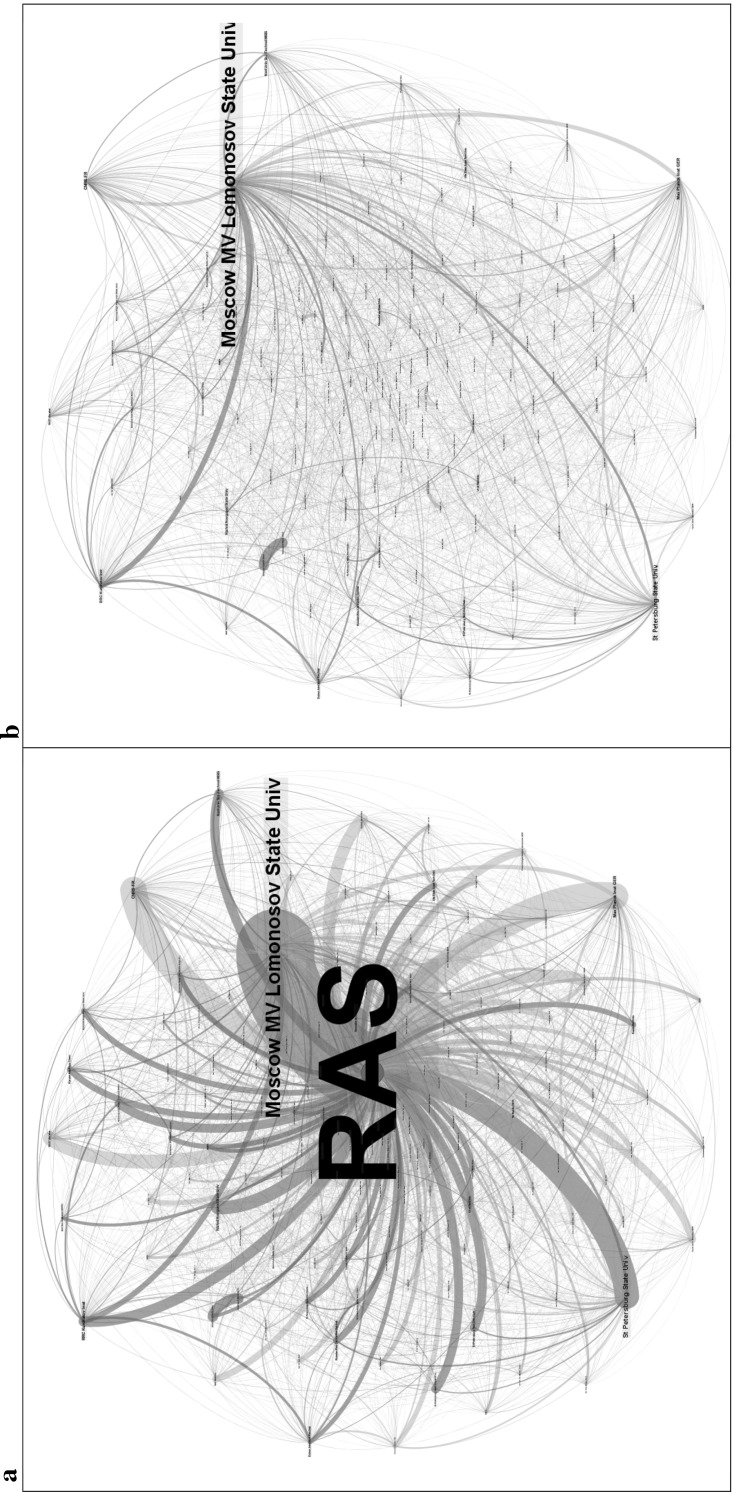
Network diagram of organisations publishing in Russian nanotechnology, 1990–2012. **a** Co-publication network of all author affiliations. **b** Co-publication network of author affiliations without RAS. *Source*: Web of Science. See text for details. *N* = 33,538 publication records. Network diagram produced with Gephi v0.82 and v0.9 software using the Force Atlas algorithm that maps organisations on the basis of repulsion (pushes organisations not co-publishing or co-publishing very small number of publications apart) and attraction (pulls organisations together based on their co-publication patterns) (Cherven 2013). We use an attraction distribution, which forces authorities to the centre of the graph. *Nodes* represent author affiliation organisations and edges represent co-publication between authors affiliated with nodes. Sizes of the nodes and node labels are proportional to the number of publications produced by that node over the entire period of 1990–2012. Only 160 organisations that have more than 50 publications are displayed due to complexity of the network diagram. *Blue colour* in nodes and edges represents international collaborating organisations and international co-publications, and the *red colour* represents Russian organisations and national collaborations. *Part a* depicts all organisations including the Russian Academy of Sciences (RAS), while *part b* is a network diagram of all national and international author affiliations without RAS. (Color figure online)

The Academy of Sciences, 15 universities and four State Research Institutes are the leading organisations in terms of publication output. Some 68 % of domestic publications are produced by the Russian Academy of Sciences and another 12 % by Moscow State University (MV Lomonosov). The top five organisations together produced 80 % of all publications in 1990–2012 (Fig. 2). While RAS is dominant overall in nanoscience publications with 68.5 % share, there is also high stratification among the 261 RAS institutes that publish nanotechnology papers. The dominant RAS institute, the Ioffe Institute of Physics and Technology, co-authored 20.6 % of all RAS nanotechnology publications and 14 % of overall Russian publications. Authors from the top ten RAS institutes wrote 56.7 % of RAS and 38.5 % of Russian nanotechnology publications. Universities have been catching up with RAS in nanotechnology publishing in the past decade. Moscow State University—the oldest university in Russia—outperformed the biggest RAS organisation in 2009–2012. Nevertheless, the domination of the Academy as a whole persists with a 62.3 % share of all Russian nanotechnology publications in 2012. A bibliometric map of co-authorships in Russian nanoscience confirms the dominant organisations and also depicts publishing interactions in Russian nanotechnology research (Fig. 3). The Russian Academy of Sciences dominates the publication landscape as a strong authority as it is located at the centre of the network graph (Fig. 3a) While there is no other authority in the map, other hubs are also minor compared to RAS. The vast majority of main national and international collaboration links go through RAS. The network of co-publishing organisations looks entirely different if RAS is eliminated from the picture (Fig. 3b). The network becomes much more scattered and some international organisations completely disappear from the network diagram as they solely collaborate with RAS.

This general outline of Russian nanotechnology research gives an initial indication of patterns and trends. The following analysis probes the legacy and the current influence of interconnected path-dependent features of the system by examining publication pathways of Russian papers, the geographical distribution of publications, institutional diffusion (intra-national collaborations) in the system, and international collaboration patterns.

### Journal gateways

There is a reported trend among Russian researchers to publish outputs in journals that are edited and published by Russian organisations (Oleinik 2012). The most prominent of these journals are simultaneously translated into English, which grants exposure to the authors without the necessity to adapt to the rules of ‘foreign’ peer review. The Russian Academy of Sciences assumes the role of a ‘gatekeeper’ as it publishes the majority of these journals, thus possessing the ability to block domestic and international publications of nonconformist authors and research groups.

The data for journals in which Russian co-authored publications can be found, is available for 32,844 publications, which constitutes 97 % of the data. The majority of Russian publications in English were published in translated journals. The time of publication (simultaneously or with a lag) and the translation (done by the author or by the English-language publishing house) vary from journal to journal. Of the top ten journals, containing more than one quarter of all publications, seven are translated versions of Russian journals (Table 2). Of the top twenty journals, 15 are translated (35.3 % of the dataset). The translated versions of Russian journals are identified not by the publishing body (the rights to publish in many cases are owned by Springer), but by the contents of the journal and the editorial board. We visited the Russian language web pages for selected top translated journals. In most cases, there is an explicit statement that the Russian and the English versions of the paper are identical. For example, Journal of Experimental and Theoretical Physics Letters (JETP Letters), edited by V.T. Dolgopolov of the Institute of Solid State Physics RAS states that, for Russian and English versions of each article, “they are essentially the same paper” (JETP Letters 2015). The (Springer) English edition appears two months after the original Russian paper. Similarly, Springer publishes *The Physics of the Solid State*. The description on the website says “The journal Physics of the Solid State presents the latest results from Russia’s leading researchers in condensed matter physics at the Russian Academy of Sciences and other prestigious institutions” (Springer 2015). An analogous journal, called *Phyzika Tvyordogo Tela* (*The Physics of the Solid State*) is published in Russian by the Ioffe Institute in St. Petersburg (Ioffe Physical Technical Institute 2015). The Chief Editor of both journals is A.A. Kaplyanskii. All members of the editorial board are affiliated with institutions located in Russia or the former Soviet Union. The editorial boards of the both journals as well as the tables of contents of issues are identical.

**Table 2:** Top 10 journals of Russian nanotechnology, 1990–2012

Journal rank	Journal title	Publishing body	Publications	Share of all publications (%)
1	Physical Review B	APS	1595	4.86
2	Physics of the Solid State	RAS	1412	4.30
3	Semiconductors	RAS	1255	3.82
4	Technical Physics Letters	RAS	848	2.58
5	JETP Letters	RAS	828	2.52
6	Inorganic Materials	RAS	511	1.56
7	Applied Physics Letters	American Institute of Physics	510	1.55
8	Journal of Applied Physics	AIP Publishing	505	1.54
9	Journal of Experimental & Theoretical Physics	RAS	490	1.49
10	Russian Chemical Bulletin	RAS	411	1.25

*Source* Web of Science. See text for details. *N* = 32,844 publication records

This system implies that a paper, after having undergone domestic peer-review, is published in the English version of the journal and is then indexed by the Web of Science. Approval of the domestic academic community, therefore, becomes crucial for any Russian researcher to establish and maintain a successful publication record. The Russian Academy of Sciences, including through the Ioffe Institute of Physics and Technology, issues the majority of the top journals in the dataset. RAS assumes a gatekeeping role over publication routes of Russian publications. Editorial boards mainly consist of members of RAS. This *status quo* is grounded in history as many of these journals were founded during the Soviet Union to inform the world of the achievements of Soviet science.

After the breakup of the Soviet Union, these established publication pathways and journals have been maintained without much impetus for change. While Russian scientists can and do publish in international journals, publishing in a Russian journal with a simultaneous translation into English offers a double benefit. It is an opportunity to write in their own language and gain domestic readership and recognition, which is necessary for promotion within domestic hierarchies. Russian researchers are trained to write publications according to the well-understood criteria for the domestic peer-review. The competition for space in a Russian journal is typically smaller than the competition in a high-impact English-language journal. At the same time, a simultaneous translation into English grants international exposure. Often the publisher carries out the translation, so researchers need not be proficient in a foreign language to publish.

This may partially explain why, after an initial burst in the early 1990s, more Russian WoS publications have been coming from the translated versions of Russian journals. Until 1998 there were more contributions from Russian authors in international journals, but after that point an increasingly large amount of nanotechnology research was published in the translated Russian journals. In 2011, Russian authors published 2.74 times more publications in translated Russian journals than in international journals (1170 and 426 accordingly).

Such a trend in publication pathways has two main implications. First, for Russian researchers, domestic recognition matters more than publishing internationally. Second, the Academy of Sciences, which dominates publication gateways, can exercise various forms of influence over (translated) international, as well as domestic public nanoscience, such as lexicology, structural elements of research papers, and the scope of published research areas.

A comparison is provided by experience in China. Zhou and Leydesdorff (2006) report on the gatekeeping role of Chinese-language journals in the internationalisation of Chinese science. Initially, few Chinese-language journals were indexed by the Web of Science. However, since the enactment of reforms that tied career progression and salaries of researchers to publishing directly in journals catalogued in the Science Citation Index (SCI) in the late 2000s (Cao et al. 2013), the situation has changed, with more Chinese-based journals indexed in WoS but also an increase in Chinese researchers publishing in international SCI journals. A similar focus on SCI publication has recently been emphasised by the Russian Government (Ogorodova 2014). However, this may not lead to the same growth effect as in China as the most reputable RAS journals are already catalogued in the SCI.

### Centralisation

Russian nanotechnology research is highly centralised geographically, as well as institutionally. RAS has institutes in all 83 regions of Russia, and the 261 RAS institutes publishing on nanoscience are located in 40 of these regions. However, Moscow, Moscow Region, St. Petersburg, and Novosibirsk published the bulk of 1990–2012 Russian nanotechnology papers, contributing over 80 % of the total output. Moscow is the leader with almost 35 % of all RAS publications. Together with the Moscow Region, the agglomeration produced 45.2 % of all RAS nanotechnology publications.

**Fig. 4:**
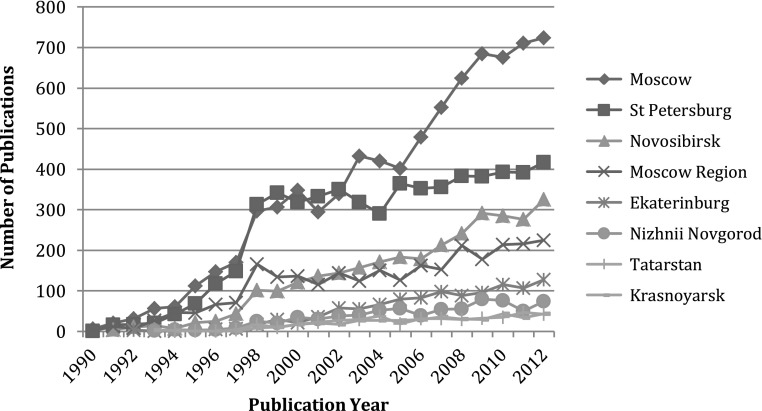
Temporal dynamics of geography of nanoscience in the Russian academy of sciences, 1990–2012. *Source*: Web of Science. See text for details. *N* = 22,794 publication records

While issues of RAS centralisation have long been observed, these trends have intensified in recent years. The Academy of Sciences’ geographical expansion in the Soviet Era, which produced a network of institutes, many remotely located with low productivity (Graham 1998), has contracted back to three main regional locations: Moscow and the Moscow Region, St. Petersburg, and Novosibirsk. In nanotechnology, RAS institutes in Moscow surged upwards in the mid 2000s, producing almost twice as many publications in 2012 as the research cluster in St. Petersburg (Fig. 4). Moscow boasts several emerging RAS institutes, while the Ioffe Institute of Physics and Technology in St. Petersburg, with several small satellites, is the other large and respected RAS in nanotechnology.

Geographical centralisation is accompanied by persistence in the locations of high quality research in Russian nanoscience. Therefore, we assess the nanotechnology-specific excellence of Russian research organisations. Bornmann et al. (2011) bibliometrically define scientific excellence in terms of rates of concentration of high performing authors who collect high numbers of citations. To investigate whether quantity translates into quality in RAS publications, we assessed the performance of the Russian domestic research system according to the criteria of (1) average citations (both nominal counts and normalised by publication years) by affiliation and (2) affiliations of the most cited researchers and publications. Out of all domestic actors, RAS publications collect the highest number of citations: 4.39 average citations per publication.^2^ Publications of public research organisations (PROs), albeit being much smaller in number, collect 3.84 citations. Universities collect on average 3.22 citations, and publications produced by corporate actors collect on average 2.44 citations. The RAS co-authored 81 out of the 100 most highly cited publications in Russian nanoscience.

The domination of RAS stretches beyond quantity of publications: it also retains the best performing personnel, and co-authors the most cited publications in Russia. Further inquiry in the peak research focuses on top performing scientists in the Russian nanotechnology in terms of the number and the quality of publications (Meyer 2006; Zucker and Darby 1995). The top 10 most cited researchers demonstrate homogeneity in the affiliation structure^3^ (Table 3). The majority of these ‘star’ scientists are, or have been, affiliated with RAS Ioffe Institute of Physics and Technology in St. Petersburg. The Institute itself has an average citation of 6.13.

**Table 3:** “Star” scientists of Russian nanoscience

Volume rank	Citation rank	Year-normalised citation rank	Author name	Affiliation(s)	Active years
1	2	8	Ledentsov, N	RAS Ioffe Physical Technical Inst	1991–2012
2	1	12	Ustinov, V	RAS Ioffe Physical Technical Inst	1994–2012
3	7	6	Alferov, Zh	RAS Ioffe Physical Technical Inst	1990–2008
4	8	69	Kop’ev, P	RAS Ioffe Physical Technical Inst	1992–2012
5	3	5	Zhukov, A	RAS Ioffe Physical Technical Inst; St. Petersburg Acad Univ RAS (since 2010 double affiliation)	1994–2012
6	6	6	Valiev, R	RAS Inst of Metals Superplasticity Problems; State Tech Univ of Aviation (since 1997)	1991–2012
7	34	14	Egorov, A	RAS Ioffe Physical Technical Inst; St. Petersburg Acad Univ RAS (2011)	1994–2011
8	51	1	Morozov, S	RAS Inst of Microelectronics Technology and High Purity Materials	1998–2012
9	14	11	Maximov, M	RAS Ioffe Physical Technical Inst	1994–2012
10	10	41	Kovsh, A	RAS Ioffe Physical Technical Inst (1997–2003); Innolume GmbH (2003–2012)	1997–2012

*Source* Web of Science. Includes only authors affiliated with domestic organisations. Year normalised citation ranking is estimated based on the proportion authors affiliated with non-Russian organisations (around two-thirds of all authors included in the dataset) *N* = 33,538 publication records

The publication activity of the most productive scientists peaked between 1998 and 2000, after which it declined. The Post-Soviet period saw the ascendance of scientists trained in the latter years of the Soviet Union. A drop in productivity coincides with the completion of the active research phase of their careers. There is a lack of new ‘rising stars’ in the system, which explains the decline of the overall performance. This data relates back to concerns of the ‘generation gap’ in nanotechnology where the average age of the researcher leans to mid-50s (Terekhov 2011).

The most productive periods of the most productive Russian nanoscientists coincides with the most productive periods of Russian nanoscience: the contribution of “star” scientists was above 9 % in 1996–2001, reaching a peak of 11.5 % in 1998. A second and smaller peak was reached in 2006, after which the decline aggravated. ‘Star’ scientists are an extremely important element of high performing national and regional science, technology and innovation systems (Heinze et al. 2009). For example, Zucker and Darby (1996) link the research, knowledge transfer and entrepreneurial activities of US ‘star’ scientists with product development, commercialisation and enterprise employment growth. While no commercialisation increase was reported after the star scientists’ outputs peaked, Russian ‘stars’ did collaborate with corporate actors on average twice as actively as RAS, where they all have been employed, in general: 8.4 % versus 4.4 %. One of the ‘star’ scientists left RAS to be employed in a German company, but the rest have demonstrated little or no mobility throughout their careers.

The network analysis also reinforces the bibliometric results presented above (Table 4). The network of co-publishing author affiliation organisations is very dense. The most direct way of measuring this is the graph density measure which indicates the level of co-publications relative to the total possible value (Cherven 2013). In terms of individual organisations, RAS is central to the network in all periods, according to a number of different measures. At the same time, RAS collaborates to a very limited degree with other research organisations in the domestic system. The most straightforward measure for this is normalised degree centrality, which shows number of connections from RAS as a percentage of all possible connections. This measure has been around 70 % in all three periods, which implies that RAS contributed to around 70 % of all co-publications. Similarly, betweenness centrality for RAS (i.e. how often RAS lies on the shortest path between two other nodes) is also around 70 % and is stable across the three periods of our study.

**Table 4:** Institutional diffusion of the Russian research output (Publications as percentage of total publications by an organisation)

	RAS (%)	Universities (%)	PROs (%)	Corporate (%)
Single author	5.9	5.4	5.3	5.2
Multiple authors (of which)	94.1	94.6	94.7	94.8
Single organisation only	26.3	21.9	16.2	9.2
With organisations of the same type only	6.8	4.4	1.6	0.6
With other national organisations only	19.0	30.8	31.4	54.8
With foreign organisations	42.0	38.7	46.1	31.0

*Source* Web of Science. See text for details. *N* = 33,538 publication records

### Institutional diffusion

Increasing attention has been given to the growth of inter- as well as intranational scientific collaboration networks in recent years (Glänzel and Schubert 2004). To explore institutional collaboration and associated knowledge exchange and diffusion in the Russian case, we investigate (1) whether each organisation preferred to publish on its own; (2) if research was done through the collaboration of authors in one organisation; (3) whether the organisation engaged in collaborative activities with other organisations of the same type; (4) if organisations preferred to collaborate nationally; and (5) whether organisations preferred to collaborate internationally.

The results of this analysis demonstrate various patterns of domestic collaboration (Table 5). For instance, corporate publishers rely on collaborations with other types of domestic research organisations: in collaborated papers, they demonstrate very low rates of collaboration within the organisation, or with other corporate actors, publishing over a half of papers with domestic research organisations. An asymmetric relationship among the system actors once again reflects the institutional domination of RAS. Collaboration links between the Academy of Sciences and other institutions are weak. About two-fifths of academic publications are written either by a single author, or by a group of authors within RAS, and only 19 % are collaborated with other Russian organisations than RAS. An international orientation is evident for PROs: over 46 % of publications are internationally collaborated, but only 1.5 % of publications are collaborated with other domestic PROs. Higher rates of international collaboration among PROs reflects their highly specialised nature: mainly nuclear or particle physics, fields where such strategies are a norm. University organisations stand in the middle and have larger share of nationally collaborated publications than the Academy or PROs. Leading RAS institutes have the most pronounced international orientation. For instance, the Landau Institute of Theoretical Physics in the Moscow Region has collaborated in only 11.6 % of its publications with domestic actors, preferring to search for international partners.

**Table 5:** Network analysis of Russian nanotechnology publications

Measure	Definition	Transitional period (1990–2004)	National nanotechnology initiative (2005–2007)	Recent period (2008–2012)
Number of nodes	Number of author affiliation organisations	2076	1623	2670
Number of edges	Number of connection (i.e. co-publications) between nodes	7990	6503	12,421
Network diameter	Maximum number of connections (i.e. co-publications) required to traverse the graph	7	6	6
Graph density	Level of co-publications relative to the total possible value	0.004	0.005	0.003
Average path length	The shortest possible path between all organisations	2.45	2.46	2.48
Connected components	The number of weakly connected components	111	50	95
Average clustering co-efficient	Average level at which the organisations are grouped together	0.73	0.75	0.74
Betweenness centrality (RAS)	How often RAS lies on the shortest path between two other nodes	0.70	0.74	0.68
Normalised degree centrality (RAS)	Number of connections from RAS divided by all possible connections	0.69	0.70	0.69

*Source* Web of Science. See text for details. *N* = 33,538 publication records

Network analysis also supports our finding that institutional diffusion is very limited. The average clustering co-efficient (i.e. average level at which the organisations are grouped together) is low (around 74 % of all organisations cluster together) and it is sustained throughout the period. Similarly, the number of weakly connected components (around 5 % of all nodes) has not changed over time (Table 5).

### Internationalisation of research

Russia has a Eurocentric orientation in its international nanotechnology scientific collaboration patterns: Russian authors published 2.3 times more nanotechnology papers with the leading European countries—Germany, France and the UK—than they did with the USA in 1990–2012. Germany is the leading research partner for Russian authors (Table 6). At the same time, in concert with previous research on international scientific cooperation (Kozak et al. 2015), we found that Russia maintains the network of research links with countries of the former Soviet Union and the Warsaw Pact.

**Table 6:** Shares of ICP and average citation rate of Russia’s main collaboration partner countries, 1990–2012

Country	Germany	USA	France	UK	Japan	Sweden	Italy
% ICP	28.6	18.9	11.7	8.0	6.8	4.8	4.5

Country	Ukraine	Poland	Spain	Netherlands	Belarus	Finland	South Korea
% ICP	4.1	3.4	3.4	3.3	2.9	2.7	2.2

*Source* Web of Science. See text for details. *N* = 14,440 publication records. *ICP* internationally collaborated publications

The initial analysis of collaborated publications highlights the prevalence of domestic collaborations over international collaborations. This is also congruent with the findings on the general Russian scientific collaboration pattern beyond nontechnology (Marshakova-Shaikevich 2010). Among the internationally collaborated publications (ICP), the share of European countries (Western, Central and Eastern Europe) is the highest—about 75 %—followed by North America (20 %) and Asia (16 %). 8.3 % of ICP were collaborated with the countries of the Commonwealth of Independent States (CIS), which comprises nations of the former Soviet Union, with an exception of the Baltic states—Latvia, Lithuania, and Estonia; and Georgia since 2009.

In terms of research performance, nanotechnology publications with only Russian authors are cited on average 2.5 times per publication. The average number of internationally cited publications is 4.33 times: international collaboration increases average citation by a factor of 170 %.

**Fig. 5:**
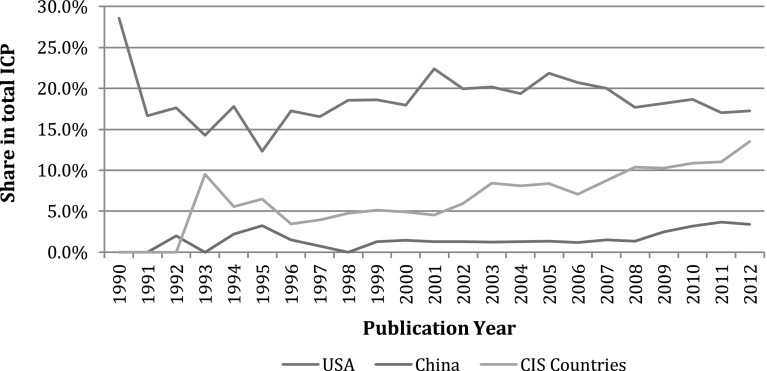
Dynamics of Russia’s collaboration in nanotechnology with the USA, China and the CIS countries. *Source*: Web of Science. See text for details. *N* = 33,538 publications

Europe has remained the largest collaboration partner of Russia throughout the post-Soviet period. The United States has been actively expanding its collaboration links with the developing countries and became China’s largest collaboration partner in nanotechnology (Shapira and Wang 2010; Tang and Shapira 2011). With regard to this trend, the nature of Russia-US collaboration patterns has fluctuated between 18 and 22 % share in the total ICP output (Fig. 5). Russia’s collaboration with China, the largest national publisher in nanotechnology, has also been very weak throughout the observation period. Furthermore, the links of Russia with CIS countries have been strengthening in recent years. Former Soviet countries have experienced extensive brain drain to Europe, America and Russia, and as well as systemic problems across the region. Such practices imply the existence of older networks in the current system, and research takes place through these interactions. At the same time, average citation rates for papers co-written with CIS based authors (8.3 % of all ICP) are significantly lower than for other countries with the same collaboration intensity.

Qualitative insights from the interviews we conducted to complement the bibliometric data hint that the international collaboration patterns of Russia mirror old path dependencies. The Russian Academy of Sciences maintained a network of research institutes across the post-Soviet countries, so institutional and personal links sustained post-breakdown. It is also known that Russia experienced extraordinary rates of brain drain throughout the post-Soviet period. The largest shock was in the 1990s, when a large number of first generation researchers left the country, taking up positions in Western Europe and North America (Borjas and Doran 2014; Ganguli 2014). Yet, Russian researchers abroad often maintain links with colleagues at home. As one of our interviewees with expertise in the area of international scientific collaboration noted, many of Russia’s collaborations with Western Europe occur between Russian scientists and their émigré colleagues.

## Discussion

This study highlights four major path-dependent structural features of Russia’s research system that have significant impact on its nanotechnology research outputs in the post-Soviet period. Previous studies have tended to focus on underfunding, deteriorating equipment, brain drain and other factors that, without doubt, are important in understanding the circumstances of research in Russia. In this paper, using bibliometric analysis and the case of nanotechnology, we draw attention to other less explicit, but nonetheless important, underpinning factors that frustrate successful implementation of science and technology policies and may weaken returns on research investment. The next round of Russia’s science policy reforms will have to consider a broader range of institutional configurations and established practices that require revision beyond direct monetary investment and infrastructure building. Some of the path-dependent features of Russia’s science system highlighted in this paper have remained unchanged since the breakup of the Soviet Union.

The findings of this research have particular significance for policymaking in a country, such as Russia, with entrenched autocratic science management approaches (Alferov 1996; Graham 1998). There are multiple factors in the structure of Russian nanotechnology research that limit not only the outputs of the system, but also have impact on its ability to change. Inertial elements of the science system, such as the privileged position of RAS, need to be addressed. Some of the path-dependent rigidities of Russian science were tackled in the recent round of science policy reforms, yet others remain unchanged.

The Russian Government initiated its latest round of large-scale science and education system reforms in the late 2000s, which coincided with the first failings of the technology-based big funding programmes, mainly, the ambitious nanotechnology initiative. These reforms have so far taken two broad directions: the reform of research-performing organisations and the reform of the funding system.

State priorities shifted from supporting academic research to the development of a network of research-intensive universities. In the first instance, the Federal Universities initiative (2009) funded selected regional universities generously, as well as other educational bodies. The National Research Universities initiative (2009) allocated large blocs of funding to a selected cohort of best-performing universities in Russia to facilitate the shift from teaching-based profile to a research-based profile. The ‘1000 laboratories’ programme and the ‘Mega-grant’ programme (Dezhina 2010; Dezhina and Ponomarev 2013) were initiated to develop domestic research by engaging leading international scientists in selected universities.

In the second instance, the Russian Academy of Sciences underwent a major transformative process. The new system seeks to optimise academic research by increasing its funding and allocating it on a competitive, not distributive, basis. A new civil agency manages the property of RAS since 2013, its head appointed by the government. RAS was merged with the Medical and Agricultural Academies and since 2015 is asked to elect young (below 50 years of age) Academy professors (Presidium RAS 2015).

RAS reform was controversial and not welcomed by the Academy community. There were concerns that the pace of research would be hindered by the mismanagement of academic property by non-professionals, and also because the reforms did not address other pressing problems within RAS (Clark 2013; Stishov 2013; Yablokov 2014). Although academicians are reluctant to enact their new ‘science advisory’ functions and join in with the restructuring of the institutes, RAS reform does not seem to threaten its monopoly on science (Nature 2013). Our research indicates early signals of emergence of alternative clusters of nanotechnology research and centres of excellence, but RAS has stayed dominant throughout the observation period, and this ‘domination cap’ seems to be what truly needs to be targeted by the reform.

Anther block of science policy reforms targeted the shift from distribution to competition in the allocation of science funding. While science priorities are still set centrally at the Federal level, the Russian Government employs advanced methods, such as technology forecasting and foresight, to determine emerging research areas and markets (Sokolov 2013). A system of competitive funding allocation was enhanced when the Russian Science Fund was founded in 2013. It manages unprecedented amounts of financial support, reaching 18.7 bln Rub in 2016 (RSF 2016). The most recent effort to enhance science enacts assessment of research performance based on key performance indicators (Government of Russia 2009). The core of these indicators is publication of research articles in SCI based journals.

The efforts of the government to enhance university research, establish the system of competitive science funding and reform Academic institutes are expected to further contribute to a decentralisation of the national research system and the emergence of new centres of excellence. However, these tools poorly address the path-dependent practices embedded in the system, as identified by this research. For instance, existing science policies do not target the weak intranational collaboration structures and isolationist stances of top research organisations. Supporting strong players has officially become a focus of Russia’s regional science and innovation policies (Vercueil 2014), so further regional centralisation should be expected. The enactment of key performance indicators, expected to boost Russian scholarship indexed in the Web of Science, does not decrease the importance of journal gateways. On the contrary, these gateways become more important because they offer an opportunity for Russian researchers to publish in the WoS without actually competing internationally but only going through domestic peer review. With regard to the internationalisation of research, Russia is reported to be the only one of the BRICS countries that did not adjust its research priorities to those of the developed countries (Finardi 2015). The further development of Russia’s international science links and, especially, international funding, has become even more difficult in the current geo-political climate (Pokrovsky 2015).

## Conclusions

Russia’s focus on nanotechnology has not fulfilled the initial aim of scientific, technological and economic catch-up with leading countries. Instead, this effort revealed deeply rooted inefficiencies within the national system of science and innovation, even in the areas of physics and carbon nanostructures that are, thought to be internationally competitive. After its underperformance was recognised, nanotechnology fell out of political grace with Russian policymakers, and priority nanotechnology science funding programmes were not continued beyond 2012. In new initiatives, the emphasis is on other strategic emerging areas, such as biotechnology (Agency of Strategic Initiatives 2016), but in these areas the Russian Academy of Sciences and the Russian Academy of Medical Sciences are again the dominant organisations, and multiple similar issues with implementation have been reported (Roffey 2010).

Path-dependent features, such as the English translation of prominent Russian journals and ‘international’ émigré collaborations, complicate assessments of the scale, quality and depth of Russian scientific research in nanotechnology. Internationalisation and globalisation are increasingly sought and incentivised in leading research systems (The Royal Society 2011). However, secluded and largely national research has to a large extent remained a dominant form in the configuration of scientific research in Russia since the breakup of the Soviet Union. This pattern may well be reinforced in the light of recent political and economic events in Russia and between Russia and its neighbours.

On a more general level, this paper illustrates the role of path dependence in inhibiting the transformation of a research system. Path dependence is revealed in informal and formal institutions, such as unchanged international links, scientific outlets and, most significantly, in the Russian Academy of Sciences. Often, the long-term strength of a system lies in its capacity to effectively change itself by transforming its fundamental building blocks, i.e. its constituent institutions. In case of Russia, the fundamental institutions have maintained core rigidities that are locked into the research system.

It is unrealistic to expect that the insertion of new financial resources will in itself lead to changes in scientific relationships and practices in the presence of strong path dependencies, the root causes of (under)performance in an established research system usually run deep, reaching beyond current circumstances and resources. Additionally, formal and informal institutions matter in the performance of a research system. These institutions have legacies, which influence their capacity to help or hinder change. Basic incentive structures or forced regulatory reforms, which reinforce path-dependent behaviour, often fail to create significant change in these institutions and subsequently the systems that they govern. Thus, a pre-condition for the long-term effectiveness of policy is a comprehensive understanding of path dependencies and their impact on a system. Policies that only target superficial and short-term behaviour, ignoring path dependencies, almost always fail to make impact due to the self-adjusting nature of institutions that resist structural change. For instance, it is difficult to elevate the role of research universities *vis*-*à*-*vis* the RAS by simply increasing resources given to the former and at the expense of the latter. Additionally, such policies neeed to be accompanied by persistent and effective mechanisms to redistribute science governance authority, including consideration of the power of RAS over domestic and international publication opportunities.

We recognise that the bibliometric database used in the study, with its emphasis on English as the language of publication, is a source of potential limitations in the explanatory mechanisms of this research (Moed 2002). In this paper we state that domestic recognition and production seems to be more important for Russian researchers than international recognition, and in recent years—increasingly so. However, the most reputable Russian nanoscience research is still published in English using the translated journals edited by RAS, which reduces the language bias. There are other limitations in the research, which should be recognised. This includes the focus on nanotechnology. This provides a useful case to examine frontier research, but at the same time caution needs to be exercised in generalising findings from this interdisciplinary field to the level of the whole research system and in inferring causality between publication outputs and system dynamics. In compensation, we used the qualitative material collected in Russia to triangulate some of the observations made in this paper. Finally, we were unable to assess Russia’s military nanotechnology research progress, which constitutes a considerable component of nanotechnology funding and outputs.

## Footnotes

1 For discussion of technical and bibliometric definitions of nanotechnology, see Porter et al. (2008), and Arora et al. (2013).

2 The addition and removal of English and Russian journals in the database in the 1998–2001 period may have caused errors with citation counts. While this may have affected total numbers, proportions and shares are not affected and are comparative.

3 The most highly cited Russian scientists are the ones that collaborated with colleagues at the University of Manchester in the award-winning research on graphene in Science (Novoselov et al. 2005). This publication has 3541 citations. To include this exceptionally highly cited publication into the data would overshadow the underlying pattern of Russian nanotechnology performance, so this publication is excluded from the citation analysis.
